# The prevalence of hepatitis B virus among HIV-positive patients at Kilimanjaro Christian Medical Centre Referral Hospital, Northern Tanzania

**DOI:** 10.11604/pamj.2017.28.275.11926

**Published:** 2017-11-28

**Authors:** Tasilo Kamenya, Damian Jeremia Damian, James Samwel Ngocho, Rune Nathaniel Philemon, Michael Johnson Mahande, Sia Emmanueli Msuya

**Affiliations:** 1 Kilimanjaro Christian Medical University College (KCMUCo), Moshi, Tanzania; 2 Department of Community Medicine, KCMC Hospital, Moshi, Tanzania; 3 Department of Epidemiology and Biostatistics, Institute of Public Health, KCMUCo, Moshi, Tanzania; 4 Department of Paediatrics, KCMC Hospital, Moshi, Tanzania; 5 Department of Community Health, Institute of Public Health, KCMUCo, Moshi, Tanzania

**Keywords:** Hepatitis B virus, Human Immunodeficiency Virus, hepatitis B virus, co-infection, Tanzania

## Abstract

**Introduction:**

Human Immunodeficiency Virus (HIV) and hepatitis B virus are prevalent infections in sub-Saharan Africa, but information on the prevalence of co-infection is limited. This study aimed to determine seroprevalence and risk factors for hepatitis B virus infection among people living with HIV receiving care and treatment at Kilimanjaro Christian Medical Centre Referral Hospital in northern Tanzania.

**Methods:**

This was a cross-sectional study conducted from March to June 2015 among people living with HIV (PLWHIV) aged 15 years and above attending the Care and Treatment Clinic for routine care at Kilimanjaro Christian Medical Centre. Systematic sampling was used to select the study participants. Information on socio-demographic data, sexual behaviour and medical history were collected using a questionnaire. Hepatitis B surface antigen was diagnosed using a rapid test. Descriptive statistics were used to summarize the data.

**Results:**

A total of 300 PLWHIV consented to participate in this study, of whom 62% were female. Their ages ranged from 15-75 years, with a median age of 46 years (IQR of 39-53 years). The seroprevalence of hepatitis B surface antigen among people living with HIV was 2.3% (n=7/300). A history of blood transfusion was the only factor associated with hepatitis B surface antigen infection, while other socio-demographic and clinical factors showed no association.

**Conclusion:**

Hepatitis B virus infection is infrequent among PLWHIV in this setting. Despite the prevalence, we recommend routine screening for hepatitis B surface antigen and other hepatitis B virus markers among PLWHIV in order to tailor antiretroviral regimens against hepatitis B virus.

## Introduction

Chronic hepatitis B virus (HBV) infection is defined by the World Health Organization (WHO) as having positive hepatitis B surface antigen for at least 6 months [1]. HBV affects 240 million people globally, with the highest prevalence in East Asia and sub-Saharan Africa (SSA) [1]. It is the 10th leading cause of death worldwide. Approximately 686,000 deaths per year are caused by chronic hepatitis and hepatocellular carcinoma [1-3]. SSA also has the highest number of people living with HIV (PLWHIV) globally (70% of the 36.7 million) [4]. Globally, it is estimated that 4 million PLWHIV are also co-infected with HBV. SSA is the most affected, and the region is also expected to have a high prevalence of co-infection [3, 5]. These two infections co-exist because of their similarity in risk factors and transmission mechanisms. People co-infected with HIV and HBV have lower rates of spontaneous clearance of HBV, and studies have shown that they may have a risk of developing chronic HBV infection that is 10 times higher than that of the general population [2, 6, 7]. HIV also increases the risk of liver cirrhosis among those with chronic HBV [8, 9]. The risk of liver-related mortality has been found to be 2-3 times higher in HIV-HBV co-infected patients than in HIV mono-infected patients [9-11]. However, with increasing access to highly active antiretroviral therapy and with 17 million people on Antiretroviral Therapy (ART) by the end of 2015, there has been a major reduction in opportunistic infections and improvement in life expectancy for HIV-infected individuals, even in SSA [4, 10]. Liver disease is now emerging as an important cause of morbidity in patients with HIV-HBV co-infection [12-14]. There is thus a need for clinicians and public health workers in SSA to understand the epidemiology of HBV-HIV coinfection [3, 4, 13].

Only a few studies have evaluated HBV-HIV co-infection in Tanzania. One study done in 2007 at a tertiary hospital in Dar es Salaam showed that 17% of PLWHIV had hepatitis B surface antigen (HBsAg) [15]. Another cohort study of PLWHIV that enrolled patients from various primary health clinics in Dar es Salaam from 2004-2011 found the prevalence of HBsAg among PLWHIV to be 6.2% [12]. Tanzania introduced the hepatitis B vaccination into its routine immunization schedule in 2002 and a national ART treatment programme in 2004 [16]. The regimens have changed over the years in Tanzania following WHO recommendations. In Tanzania, patients receiving ART are on different regimens depending on when the ART was initiated. Currently, routine HBV screening among HIV-positive patients is not conducted in Tanzania. However, with the 2030 goal of eliminating viral hepatitis as a public health problem [13] and the scale-up of ART in SSA, including Tanzania [4], there is a need for data on HBV-HIV co-infection, since some specific ART drugs used for HIV treatment can be interchanged to target both infections. Further, for PLWHIV who have not previously been exposed to HBV, the recommendation is to offer vaccination for HBV [2, 13]. This study aimed to describe the seroprevalence and associated factors of HBsAg among PLWHIV aged 15 years and above attending the HIV Care and Treatment Clinic (CTC), Kilimanjaro Christian Medical Centre (KCMC) Referral Hospital in Moshi Municipal, northern Tanzania.

## Methods

**Study design and site**: A cross-sectional study was conducted from March-June 2015 among PLWHIV attending the Care and Treatment Clinic (CTC) at KCMC Referral Hospital in Moshi Municipal, Tanzania. This district is one of the seven districts of Kilimanjaro Region, situated in northern Tanzania, with a population of approximately 184,292 people [17]. There are 54 health facilities in Moshi Municipal: 4 hospitals, 8 health centres and 42 dispensaries. Of these facilities, 14 offer HIV care and treatment services: 3 hospitals, 5 health centres and 6 dispensaries. KCMC Referral Hospital was selected because it is one of the facilities with high attendance of PLWHIV in the district.

**Study population and procedures:** The study population included consenting PLWHIV aged 15 years and above and attending HIV CTC at KCMC Referral Hospital for routine care. The Epicalc 2000 software (Brixton Health, UK) was used to calculate the necessary sample size. The minimum sample size required was 248 patients when the confidence level was set at 95%, the prevalence of HBsAg at 17% [15] and the margin of error at 5%, adding 15% for non-response. At KCMC Referral Hospital, the CTC services are offered on Monday, Wednesday and Friday, and approximately 60 patients attend on each day. On average, each month approximately 700 PLWHIV are served at this clinic. Systematic sampling was used to select the study participants. Using the client registry, we identified every 6th person and invited them to participate. Approximately 10 participants were enrolled every day. Three hundred and twenty PLWHIV were invited to participate, and among them, 20 patients declined participation. A questionnaire was used to collect information from the eligible patients. The questionnaire was composed of questions on socio-demographic data, sexual practices and medical history. Medical files were reviewed to collect information on their current cluster of differentiation (CD4) count and type of ARV drugs used. After the interviews, venous blood was collected in the red top bottle for the diagnosis of hepatitis B surface antigen. The samples were placed for a few minutes and then centrifuged at 3000rpm for 5 minutes to obtain clear serum for the serological analysis of HBsAg. Hepatitis B surface antigen (HBsAg) was detected using the rapid test Bioline (Biotech Ltd, Dartford, UK). The test has been shown to have a sensitivity and specificity of 99.7% and 99.3%, respectively, when performed according to information from the manufacturer.

**Data analysis:** The data were cleaned and checked for completeness and consistency before analysis. Analysis was conducted using the Statistical Package for Social Sciences (SPSS) version 16 software (IBM, USA). Descriptive statistics (mean or median with their respective measures of dispersion for continuous data and proportions for categorical data) were used to summarize the data. Chi-square or Fisher’s exact tests were used to test the difference between groups. The strength of association between HBsAg and risk factors was assessed using odds ratios (ORs) with its respective 95% confidence interval. P-values less than 0.05 were considered to be statistically significant. Ethical approval was obtained from the Kilimanjaro Christian Medical University College Research and Ethical Committee. Permission to conduct the study was also sought from the Office of the Executive Director of KCMC Referral Hospital. The clients approached were informed about the purpose of the study, and written consent was obtained from each participant prior to enrolment in the study. For illiterate individuals, a thumb print was used as a signature. For patients under 18 years, consent was obtained from the patients and from parents or guardians. Those found with HBV infection were informed of the results and referred to a CTC physician who was also informed about their HBsAg-positive status.

## Results

A total of 320 PLWHIV were approached and 300 accepted, giving a response rate of 94%. The reasons for refusal to participate were poor physical health, lack of study benefits, and lack of time. The age of the 300 enrolled participants ranged from 15-75 years, with a median age of 46 years (IQR of 39-53 years). More than half of the participants (n=187) were female (62.3%), had primary education (n=167, 55.7%) and were farmers or small traders (n=206, 68.7%). A history of blood transfusion and tattooing was reported by 7.0% and 0.7% of the participants, respectively (Table 1). Most of the 300 PLWHIV were on ART treatment (99.0%), and 44.7% were on a Tenofovir- (TDF-) containing ART regimen. The median CD4 count was 453 cells/mm^3^ (IQR: 377-565). The prevalence of HBsAg infection among the 300 PLWHIV was 2.3% (n=7). Six of the HBsAg positive patients were males and one was female. The association between risk factors and HBV infection is shown in Table 2. PLWHIV with a history of blood transfusion had a higher prevalence of HBsAg (13.0%) than non-transfused people (1.4%). This difference was significant (OR=10.24, 95% CI: 2.14-48.93). A history of tattooing was also associated with HBsAg positive results; patients with no history of tattooing had 96% lower odds of being HBsAg positive than those who had a history of tattooing (OR=0.04, 95% CI: 0.01-0.19). Age, sex, marital status, number of sexual partners, history of injections, and CD4 count were not associated with positive HBsAg.

**Table 1:** Sociodemographic and clinical characteristics of the participants (N=300)

Variables	N (%)
**Age (years)**	
15-24	33 (11.0)
25-34	18 (6.0)
35-44	68 (22.7)
45+	181 (60.3)
**Marital status**	
Single	61 (20.3)
Married/Co-habiting	139 (46.3)
Divorced/Widowed	60 (33.4)
**Number of sexual partners**	
< 2	32 (10.7)
3+	268 (89.3)
Alcohol intake	50 (16.7)
History of intramuscular injection	248 (82.7)
History of blood transfusion	23 (7.7)
History of tattooing	2 (0.7)
On ARV	297 (99.0)
**Time starting ARV (n=297)**	
1995-2004	48 (16.2)
2005-2010	176 (59.3)
2011-2015	73 (24.6)
**Type of ARV regimen (n=297)**	
AZT/3TC and EFV	62 (20.7)
AZT/3TC and LPVr	24 (8.0)
ABC/3TC and LPVr or ATVr	77 (25.7)
TDF/3TC and EFV	26 (8.7)
TDF/3TC and NVP	108 (36.0)
**Current CD4 count (cells/mm^3^)**	
˂ 200	13 (4.3)
200-350	51 (17.0)
˃ 350	236 (78.7)

**Table 2:** Risk factors for HBsAg among 300 HIV-positive patients in Moshi, Tanzania

		**HBsAg positive**	**P-value**
**Factor**	**N**	**n (%)**	
**Alcohol intake**			
No	250	4 (1.6)	
Yes	50	3 (6.0)	0.093
**History of blood transfusion**			
No	277	4 (1.4)	
Yes	23	3 (13.0)	0.011
**History of intramuscular injection**			
No	52	1 (1.9)	
Yes	248	6 (2.4)	0.829
**History of tattooing**			
No	298	6 (2.0)	
Yes	2	1 (50.0)	0.046

## Discussion

In this study, the prevalence of HIV/HBV co-infection among patients aged 15 years and above was 2.3%. The prevalence of HIV/HBV co-infection observed was lower than that of two studies in Dar es Salaam, Tanzania, which showed a prevalence of 17% and 6.2% [12, 15]. The prevalence of co-infection was also lower than that shown in studies done in the Ivory Coast and Nigeria (13.4% and 7.9%, respectively) [18, 19]. Among PLWHIV in South Africa, the HBsAg prevalence was 7.1% and 7.0% among women attending CTC care [20,21]. In Uganda, this prevalence was 16.9% [22]. The difference in prevalence reflects a wide variation of HBsAg among PLWHIV in various SSA countries. Considering the epidemiology of hepatitis B virus infection (HBsAg) in Tanzania, the studies conducted showed marked variation between populations and regions. In Dar es Salaam, Tanzania, Matee et al (2006) reported a prevalence of 8.8% among blood donors, Nagu et al (2008) reported a prevalence of 17% among PLWHIV and Rashid et al (2014) observed an HBsAg prevalence of 3.9% among women attending the antenatal clinic at Muhimbili National Hospital [15, 23, 24]. In Moshi Urban, northern Tanzania, an HBsAg prevalence of 4.2% was observed among women of childbearing age in 2006 [25]. The relatively low seroprevalence of HBsAg found among HIV-positive patients in 2015 in Moshi may reflect that sexual transmission is not the major route of HBV infection in this setting. In a metropolitan city such as Dar es Salaam, the high prevalence of co-infection is partly due to illegal drug injections.

Additionally, the background prevalence of hepatitis B virus might contribute to the observed variation. Alternatively, the prevalence may be explained by the routine hepatitis B vaccination that was introduced into the national routine immunization programme in 2002. The vaccine may have lowered the transmission in the general population, which would also be reflected in PLWHIV. HBsAg screening is not done routinely among HIV-positive patients in Tanzania. Even with the relatively low prevalence of 2.3% found in this study, it is important to consider strategies to routinely screen for HBsAg, since individuals found to be HBsAg-positive will need ART regimens that include two drugs that also target the hepatitis B virus [1-3]. Benhamou et al (1999) and Gu et al (2015) have shown that if lamivudine (3TC) or emtricitabine (FTC) are used as the only HBV-active agents in ART among HIV/HBV co-infected patients, there is a higher risk of developing HBV resistance [26,27]. It is recommended that for HIV/HBV co-infections, a combination of tenofovir (TDF) with either 3TC or FTC in the ART regimen should serve as the first line of treatment [2, 10, 27]. Only two of the seven HIV/HBV co-infected patients in this study were on an ART regimen containing TDF and 3TC. The others were on an ART combination containing 3TC as the only drug effective against HBV. Furthermore, those found to be HBsAg-negative and who had never been exposed to hepatitis B virus infection should still be offered immunization [1,2]. This study found an association between HBsAg and a history of blood transfusions. However, the precision of the estimate was low because of the wide confidence interval. In Tanzania, the National Blood Transfusion Service (NBTS) was established in 2004 with the aim of ensuring the availability of safe blood and blood products for transfusion. The NBTS routinely screens voluntarily donated blood for transfusion for associated infections, including HIV, hepatitis B and C and syphilis [28]. More data are required from larger studies to explore this association. A limitation of this study is that we might have failed to show associations between HBsAg and several risk factors due to the low number of HBsAg-infected individuals. To address this shortcoming, we suggest that future studies recruit more clients. Due to financial constraints, we did not check for other markers that show HBV infectivity, such as HBeAg or antibody markers, as well as for anti-HBc or anti-HBc IgM.

## Conclusion

The prevalence of HBsAg found (2.3%) is considered to be moderately endemic in PLWHIV in this setting. The presence of HIV-HBV co-infection requires antiretroviral therapy that also targets hepatitis B virus infection; hence, routine screening of PLWHIV for HBsAg should be considered so that proper treatment can be offered to co-infected individuals in order to improve quality of life and reduce morbidity.

### What is known about this topic

- Individuals co-infected with HIV and HBV have lower rates of spontaneous clearance of HBV;
- Those co-infected are at a risk of developing chronic HBV infection. The risk of chronic HBV infection is 10 times higher among PLWHIV compared to the general population;
- The risk of liver-related mortality has been found to be 2-3 times higher in HIV-HBV co-infected patients than in HIV mono-infected patients.

### What this study adds

- Despite the practice of screening all blood and blood products for HBV before transfusion, we still found an association between history of blood transfusion and HBV. There is a need to use highly sensitive screening tests to reduce the transmission;
- Despite tattooing being uncommon practice in the area, this study provides evidence for the association between tattooing and HBV infection. There is a need to create public awareness to address the risk associated with this practice.

## Competing interests

The authors declare no competing interests.

## References

[1] World Health Organization Hepatitis B: fact sheet no 204.

[2] Centers for Disease Control and Prevention (2009). Guidelines for Prevention and Treatment of Opportunistic Infections in HIV-Infected Adults and Adolescents: Recommendations from CDC, the National Institutes of Health, and the HIV Medicine Association of the Infectious Diseases Society of America..

[3] World Health Organization Monitoring and evaluation for viral hepatitis B and C: recommended indicators and framework.

[4] United Nations Programme on HIV/AIDS (2016). Global AIDS update.

[5] Barth RE, Huijgen Q, Taljaard J, Hoepelman AI (2010). Hepatitis B/C and HIV in sub-Saharan Africa: an association between highly prevalent infectious diseases, a systematic review and meta-analysis. Int J Infect Dis..

[6] Gatanaga H, Yasuoka A, Kikuchi Y, Tachikawa N, Oka S (2000). Influence of prior HIV-1 infection on the development of chronic hepatitis B infection. Eur J Clin Microbiol Infect Dis..

[7] Brito M, Alhyraba M (2008). Hepatitis B in patients coinfected with HIV. Hosp Physician..

[8] Hoffmann CJ, Thio CL (2007). Clinical implications of HIV and hepatitis B co-infection in Asia and Africa. Lancet Infect Dis..

[9] Thio CL, Seaberg EC, Skolasky R, Phair J, Visscher B, Muñoz A (2002). HIV-1, hepatitis B virus, and risk of liver-related mortality in the Multicenter Cohort Study (MACS). Lancet..

[10] Konopnicki D, Mocroft A, de Wit S, Antunes F, Ledergerber B (2005). Hepatitis B and HIV: prevalence, AIDS progression, response to highly active antiretroviral therapy and increased mortality in the EuroSIDA cohort. AIDS..

[11] Weber R, Sabin CA, Frils-Møller N, Reiss P, El-Sadr WM (2006). Liver-related deaths in persons infected with the human immunodeficiency virus: the D:A:D study. Arch Intern Med..

[12] Hawkins C, Christian B, Ye J, Nagu T, Aris E, Chalamilla G (2013). Prevalence of hepatitis B co-infection and response to antiretroviral therapy among HIV-infected patients in Tanzania. AIDS..

[13] World Health Organization (2016). Combating Hepatitis B and C to reach elimination by 2030: advocacy brief..

[14] Fix OK, Locarnini SA, Development M, Peters MG (2007). Virology and Clinical Management of Hepatitis B and HIV Coinfection. Physician Research Network..

[15] Nagu TJ, Bakari M, Matee M (2008). Hapatitis A, B and C viral co-infections among HIV-infected adults presenting for care and treatment at Muhimbili National Hospital in Dar es Salaam, Tanzania. BMC Public Health..

[16] MOHSW (2007). Expanded Programme on immunization 2006-2010, Tanzania: Comprehensive Multiyear Plan..

[17] 1. The United Republic of TanzaniaPresident's Office Regional Administration and Local Government (2013). Moshi Municipality Council: Districts of Kilimanjaro including Moshi Municipal Council ..

[18] Attia K, Eholié S, Messou E, Danel C, Polneau S (2012). Prevalence and virological profiles of hepatitis B infection in human immunodeficiency virus patients. World J Hepatol..

[19] Tremeau-Bravard A, Ogbukagu IC, Ticao CJ, Abubakar JJ (2012). Seroprevalence of hepatitis B and C infection among the HIV-positive population in Abuja, Nigeria. Afr Health Sci..

[20] Boyles TH, Cohen K (2011). The prevalence of hepatitis B infection in a rural South African HIV clinic. S Afr Med J..

[21] Matthews PC, Beloukas A, Malik A, Carlson JM, Jooste P, Ogwu A (2015). Prevalence and Characteristics of Hepatitis B Virus (HBV) Coinfection among HIV-Positive Women in South Africa and Botswana. PLoS ONE..

[22] Baseke J, Musenero M, Mayanja-Kizza H (2015). Prevalence of hepatitis B and C and relationship to liver damage in HIV infected patients attending Joint Clinical Research Centre Clinic (JCRC), Kampala, Uganda. Afr Health Sci..

[23] Matee MI, Magesa E, Lyamuya EF (2006). Seroprevalence of human immunodeficiency, hepatitis B and C viruses and Sphiylis infection among blood donors at the Muhimbili National Hospital in Dar es Salaam, Tanzania. BMC Public Health..

[24] Rashid S, Kilewo C, Abood S (2014). Seroprevalence of hepatitis B virus infection among antenatal clinic attendees at tertiary hospiral in Dar es Salaam, Tanzania. Tanzan J of Health Res..

[25] Msuya SE, Mbizvo EM, Hussain A, Sam NE, Stray-Pedersen B (2006). Seroprevalence of Hepatitis B and C viruses among women of childbearing age in Moshi urbun,Tanzania. East Afr Med J..

[26] Benhamou Y, Bochet M, Thibault V, Di Martino V, Caumes E, Bricaire F (1999). Long-term incidence of hepatitis B virus resistance to lamivudine in human immunodeficiency virus-infected patients. Hepatology..

[27] Gu L, Han Y, Li Y, Zhu T, Song X, Huang Y (2015). Emergence of Lamivudine-Resistant HBV during Antiretroviral Therapy Including Lamivudine for Patients Coinfected with HIV and HBV in China. PLoS ONE..

[28] Mauka WI, Mahande MJ, Msuya SE, Philemon RN (2015). Factors associated with repeated blood donation at the northern zone blood transfusion centre in Tanzania. Journal of Blood Transfusion..

